# Cell-based screening strategy in the search for bioactive microbial secondary metabolites Utilization of microbial and animal cells with special functions

**Published:** 2004-02-01

**Authors:** Shigeo Iwasaki, Satoshi Ōmura

**Affiliations:** *) The Kitasato Institute, 5-9-1, Shirokane, Minato-ku, Tokyo 108-8641, Japan; **) Kitasato Institute for Life Sciences and Graduate School of Infection Control Sciences, Kitasato University, 5-9-1, Shirokane, Minato-ku, Tokyo 108-8641, Japan

**Keywords:** Microbial metabolite, cell-based screening, antimetabolite, lipid metabolism inhibitor, IL-6 inhibitor, proteasome inhibitor

## Abstract

Here we present results obtained in our group from cell-based screenings of microbial secondary metabolites using intact microbial and mammalian cells having specific functions. We present summaries of the following five categories of compounds and the strategies that were used to identify them. (1) Antibacterial agents targeting cell wall peptidoglycan synthesis as well as active agents against organisms other than bacteria were identified by a combination of *Bacillus subtilis* and *Mycoplasma* as test organisms. (2) Antimetabolites, such as herbicidal agents targeting glutamine synthase, were identified using *B. subtilis* grown on minimal medium with or without the addition of glutamine and folate inhibitors by combination of *B. subtilis* and *Enterococcus faecium*, which have distinct folate metabolic pathways. (3) Among inhibitors of lipid metabolism, acyl-CoA synthetase inhibitors were identified using two mutants of *C. lipolytica* with different deletion sites in the fatty acid metabolic pathways, HMG-CoA synthase inhibitors were identified using Vero cells cultured in the presence and absence of mevalonate, and anti-atherosclerosis agents were identified using macrophages. (4) IL-6 inhibitors were detected by a combination of IL- 6-dependent and independent murine hybridoma MH60 cells, and (5) Small molecules with TNF-like activities were identified using a mouse neuroblastoma cell line. Some advantages of such screening method and the significance of the identified compounds are discussed.

## Introduction

Numerous secondary metabolites with heterogeneous chemical structures have been isolated from microbes.1),2) It is now widely known that these microbial secondary metabolites have broad functions in nature3), 4) including interactions with living cells of bacteria, fungi, viruses, parasites, higher plants, insects and higher animals, as well as with functional biomolecules such as nucleic acids, proteins and lipids. Exploitation of bioactive metabolites started with the discovery of antibiotics, which target pathogenic microbes, but the activity profiles in recent decades have become broader and have also shifted to non-antimicrobial types of compounds. Therefore, the methods used to screen for the compounds have also changed.

Since 1965, we have undertaken comprehensive studies on bioactive substances of microbial origin by devising methods for the cultivation of microbial resources and for screening compounds. We have discovered more than 150 types of bioactive metabolites and over 330 individual substances to the year 2002.5) Among these, six are currently used as clinical medicines and eight are commercially available as biochemical reagents (Table I). Through our group’s research, the methods used to search for bioactive metabolites have been expanded from those directly targeting cells to those using enzyme systems or specific functions involved in organisms.6)–8) Many compounds have been discovered by screening methods that use living cells having or endowed with specific functions, and some of these were summarized previously.9) In this review, we present the rationale and results obtained from such cellbased screening strategies conducted in our group.

## Bacterial cell wall synthesis inhibitors and antimycoplasmal substances

The screening method described here is effective for both bacterial cell wall peptidoglycan synthesis inhibitors and antimycoplasmal substances.10),11) Among the various types of antibacterial agents, specific inhibitors of cell wall peptidoglycan synthesis were generally characterized by lower toxicity. On the other hand, antimycoplasmal agents were generally highly correlated to activities such as antifungal or antitumor activities rather than antibacterial activity.12)–14)

**Fig. 1:**
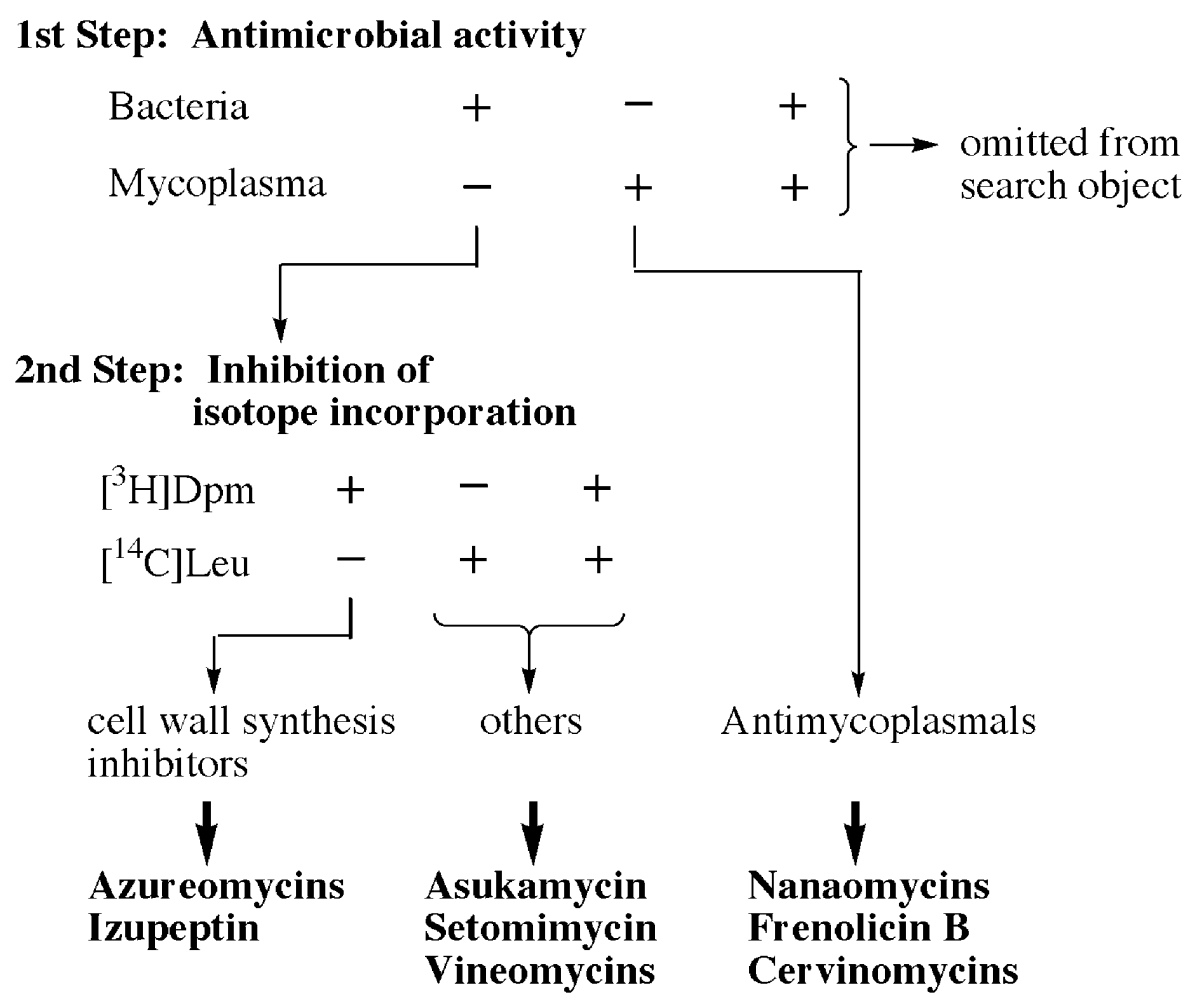
Screening procedure for bacterial cell wall synthesis inhibitors and antimycoplasmal antibiotics. Dpm, diaminopimeric acid; Leu, leucine.

The procedure consists of a two-step selection procedure. The primary step is to divide the culture broths into three groups according to their antimicrobial activities; active against *Bacillus subtilis* but inactive against *Mycoplasma*, active against *Mycoplasma* but inactive against *B. subtilis*, and active against both of them. The last group was excluded from the further screening object. The first group of culture broths were candidates for cell wall peptidoglycan synthesis inhibitors, because mycoplasma do not have cell walls.15),16) The secondary step is to screen the first group to determine if the culture broth inhibits the incorporation of [^3^H]-diaminopimelic acid ([^3^H]Dpm), a precursor of cell wall peptidoglycan, and [^14^C]-leucine ([^14^C]Leu), a precursor of protein, into the macromolecular fraction of the Dpm requiring strain of *B. subtilis*. It has been verified that the specific inhibitor of cell wall synthesis inhibits only the incorporation of [^3^H]Dpm. The general screening procedure is summarized in Fig. 1.

**Fig. 2:**
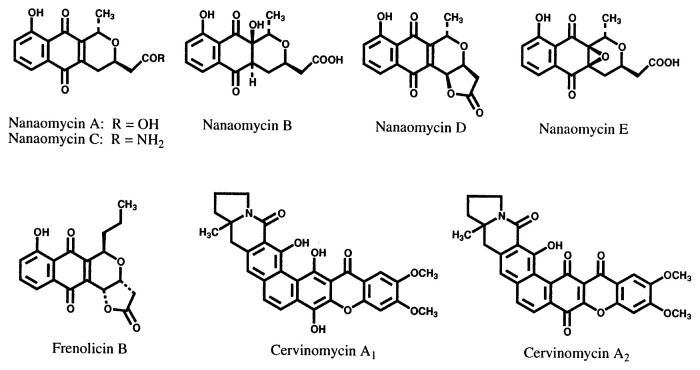
Structures of cell wall synthesis inhibitors and antimycoplasmal antibiotics.

**Fig. 3:**
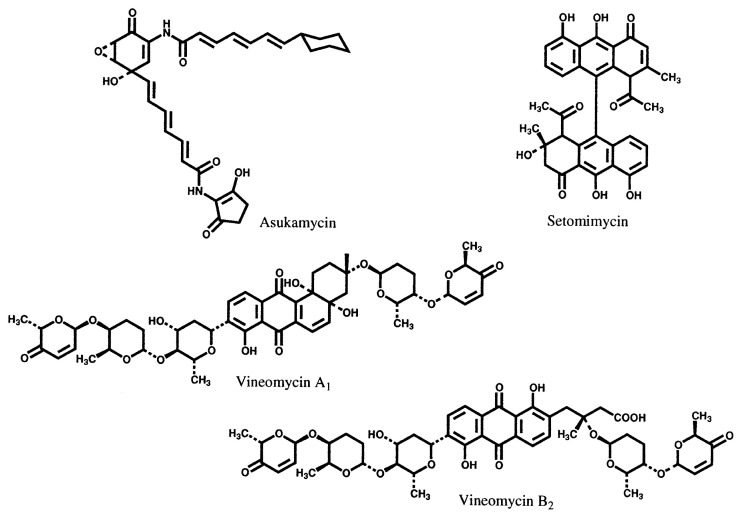
Structures of other antibacterial antibiotics obtained from the screening system shown in Fig. 1.

Based on this method new antibiotics azuleomycins A and B,10),17),18) and izupeptins A and B,19) together with several known cell wall synthesis inhibitors, were identified as new inhibitors of cell wall peptidoglycan synthesis. From the culture broths showing antimycoplasmal activity, three different types of new compounds were isolated: nanaomycins A–E which are effective against dermatomycosis of livestock 20)–25); frenolicin B, an excellent anticoccidium substance 26); and cervinomycins,27),28) which are active against anaerobic bacteria. In addition to these metabolites mentioned above, three new antibacterial substances, askamycin,29),30) setomimycin31),32) and vineomycin,33),34) were also obtained in this screening process, though they were neither specific inhibitors of cell wall peptidoglycan synthesis nor antimycoplasmals. The structures of these compounds are shown in Figs. 2 and 3.

The structures of glycopeptide antibiotics azuleomycins and izupeptin have not yet been determined. Azuleomycins were isolated from a culture broth of a new actinomycete, *Amycolatopsis azurea* nov. sp. Azuremycin B induced the lysis of growing *B. cereus* at a concentration of 10 μg/ml but did not affect resting cells.18) Izupeptins were isolated from culture broths of an actinomycete strain AM-5289. They showed potent inhibitory activity against Gram-positive bacteria. Studies on the mode of action showed that the primary target of these antibiotics is the transfer of the disaccharide peptide unit, GlcNAc-MurNac-pentapeptide, from the lipid-bound precursor to the acceptor, nascent peptidoglycan, as shown in Fig. 4.4),35)

Nanaomycins produced by *Streptomyces rosa* subsp. *notoensis* show moderate antibacterial activity and strong activity against mycoplasma as well as against *Trichophyton* spp. *in vitro* (Table II).21) Though we were unsuccessful in developing nanaomycin as an antimycoplasmal agent, a nanaomycin complex showed remarkable *in vivo* effect against dermatomycosis in cattle.36) With a single application of 100 ppm nanaomycin A, the dermatomycosis caused by *Trichophyton verrucosum* was healed in one month. After safety testing, nanaomycin A was approved as an antifungal antibiotic for veterinary use. The modes of action of nanaomycins against Gram-positive and Gram-negative bacteria have been studied. 37),38) Based on a study using marine bacterium, *Vibrio alginolyticus*, we proposed that the primary mode of effectiveness of these antibiotics is through the interference in the bacterial cell membrane function by superoxide radicals (O_2_^−^) generated in the presence of these antibiotics: The reduced form of nanaomycin A (or D) is produced by receiving electrons from respiratory flavoproteins. On oxidation of the reduced form to nanaomycin A (or D) by molecular oxygen, O_2_^−^ is produced (Fig. 539)). Though superoxide dismutase (SOD) present in the cells acts as an O_2_^−^ scavenger, the bacterium is killed due to insufficient digestion of O_2_^−^ in the presence of significant amounts of these antibiotics. Indeed, the cells having a lower level of SOD are more sensitive to these antibiotics.38)

Frenolicin B produced by a *Streptomyces roseofulvus* strain is structurally related to nanaomycin D, though their absolute stereochemistries are opposite. Cervinomycins obtained from *Streptomyces cervinus* are barely soluble in most solvents, but triacetate of cervinomycins were highly soluble and as effective as clindamycin against various anaerobic bacteria.40) Vineomycins were detected as antibacterial and antitumor antibiotics and vineomycin A_1_ was found to be identical with a *Streptomyces* metabolite P-1894B which was previously isolated as a potent prolyl hydroxylase inhibitor.41) Vineomycin B_2_ has been used as a biochemical reagent.

## Screening for enzyme inhibitors

Since many enzymes play important roles in the maintenance of homeostasis in living organisms, such enzymes or enzyme systems may be targets for selective cytotoxicities or pharmacological activities. Following the discovery of protease inhibitors from *Clostridium botulinum* and a *Penicillium cyclopium*, systematic screening of enzyme inhibitors from microorganisms started, and to date numerous inhibitors of microbial origin have been discovered. Either purified enzymes or partially purified enzyme preparations, such as cell homogenates, were used. However, this type of screening method may be too directly linked to the enzyme functions and as a result, false-positive inhibitors may often be picked up, because enzymes exhibit original activity only under specific cellular environments and, on the other hand, inhibitors of specific enzymes need to reach the targets in cells. Therefore, utilization of living cells in assays may be advantageous in the search for efficient enzyme inhibitors. The results of some of these types of screening methods developed in our group have been reviewed previously.42),43)

### (I) Antimetabolites

The term “antimetabolite” refers to an inhibitor of normal metabolism in an organism that causes damage to the organism. Various enzyme inhibitors are included among these inhibitors. The screening method for antimetabolites involves identifying substances which show activity against test organisms on minimal culture medium, but no or weak activity on natural medium or through the addition of reversants, such as amino acids, nucleic acids or vitamins, to the minimal medium. Our screening program for antiglutamates and antifolates in microbial cultures were conducted using particular bacteria as the test organisms.

#### (a) Glutamine synthetase inhibitors

**Fig. 6:**
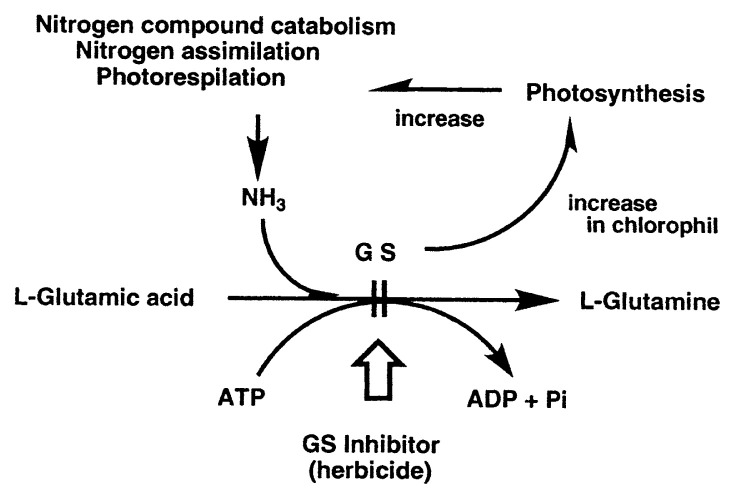
Role of glutamine synthetase in plant. GS, glutamine synthetase; ADP, adenosine diphosphate; ATP, adenosine triphosphate; Pi, inorganic phosphor.

Glutamine synthetase (GS) is the enzyme that catalyzes the amidation of glutamic acid. The enzyme plays important roles in nitrogen assimilation in bacteria and plants. In plants, GS is produced by *de novo* synthesis in chlorophyl 44),45) coupled with photorespiration,46) in order to prevent the harmful accumulation of generated ammonia by assimilation (Fig. 6). Therefore, GS inhibitor was expected to become potential herbicide. Indeed, tabtoxin isolated from *Pseudomonas tabaci* as the causative agent of tobacco wildfire disease was found to be a GS inhibitor.47)

**Fig. 7:**
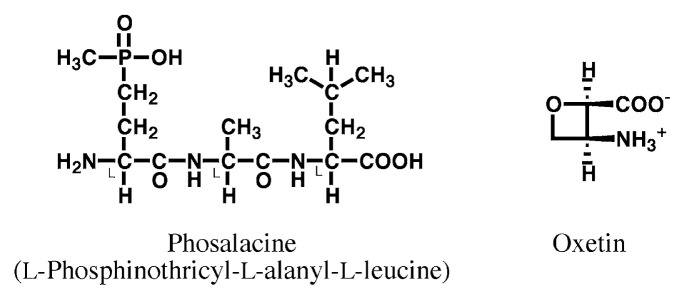
Structures of phosalacine and oxetin.

GS inhibitors were screened from the culture broths of soil actinomycetes using *Bacillus subtilis* KB-211 (PCI 219) as the test organism. The samples active against *B. subtilis* in Davis’ minimal medium but inactive in glutamine added medium were selected, as summarized in Table III. Two new compounds, phosalacine 48),49) and oxetin,50) were isolated as GS inhibitors having herbicidal activities (Fig. 7).

Phosalacine was found as a metabolite of an actinomycete strain, *Kitasatospora phosalacinea* KA-388,51) and its structure was determined to be a tripeptide comprising L-phosphinothricyl-L-alanyl-L-leucine. The compound is closely related to bialaphos, a hebicidal antibiotic composed of phosphinothricyl-alanyl-alanine. 52) Phosalacine almost completely inhibited the growth of *B. subtilis* at a concentration of 0.1 μg/ml, and the inhibition was completely overcome by the addition of 10 μg/ml L-glutamine.

Phosalacine exhibited antimicrobial activity against Gram-positive and Gram-negative bacteria and considerable herbicidal activity against alfalfa. On the other hand, the compound showed very weak inhibitory activity against glutamine synthetases from *B. subtilis* and spinach leaves, whereas phosphinothricine completely inhibited these enzymes at Ki values of 81.1 and 306 μM, respectively.48) The fact indicated that phosalacine was transformed to phosphinothricine in microbial and plant cells as indicated by bialaphos, and exhibited herbicidal activity. This example demonstrates the advantage of our strategy of searching for enzyme inhibitors using bacterial cells as test organisms. Phosalacine could not have been discovered by primary screening using *in vitro* assay, because phosalacine itself is a very weak inhibitor of the enzyme as described above.

Oxetin was isolated from a *Streptomyces* strain and found to be the first microbial product consisting of an oxetane ring. It inhibited the growth of *B. subtilis* in minimal media. Growth reversion was observed not only by L-glutamine or L-glutamic acid but also by other amino acids, such as L-isoleucin, L-methionine and L-valine. Oxetin is weakly antimicrobial, but is potently herbicidal. Oxetin noncompetitively inhibited glutamine synthetases from *B. subtilis* and spinach leaves.50)

#### (b) Antifolates

The folate metabolic pathway has been regarded as an important target for chemotherapy.53),54) Tetrahydrofolate (THF) and its derivatives are essential in cell metabolism. They are involved in the transfer of one-carbon (C1) units carried by N(5) or N(10) of THF (Table IV), and vital for the biosynthesis of purine and pyrimidine nucleotides. In fact, sulfa drugs, which inhibit the site of dihydropteroate synthase, were clinically used as main bactericides before the introduction of penicillin. Furthermore, methotrexate, trimethoprim and pyrimethamine, which act at the site of dihydrofolate reductase, have been clinically used as antibacterial, antimalarial and anticancer drugs. However, folate metabolism inhibitors of natural origin were very limited, and we tried to develop a screening method to discover new antifolates from microbial metabolites.55)

**Fig. 8:**
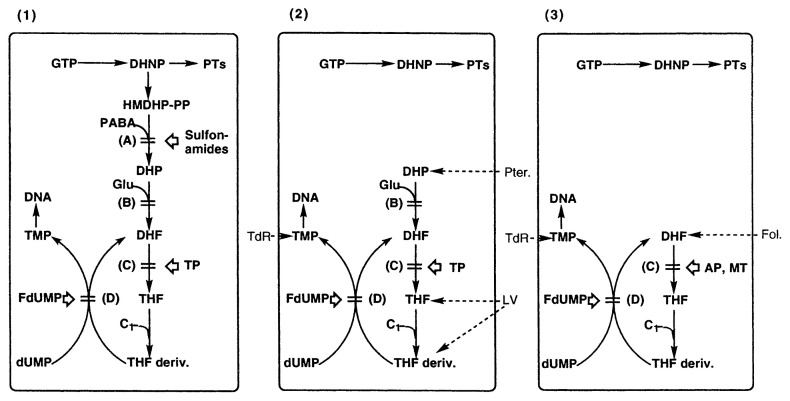
Metabolic pathways of folate in various organisms and inhibition sites of known inhibitors. (1) Usual microorganisms, (2) *Enterococcus faecium*, (3) common animal cells. Possible inhibition sites: (A) DHP synthase, (B) DHF synthetase, (C) DHF reductase, (D) TMP synthase. Abbreviations: DHNP, dihydroneopterin; HMDHP-PP, 6-hydroxymethyl- 7, 8-dihydropterin pyrophosphate; DHP, dihydropteroate, DHF, dihydrofolate; THF, tetrahydrofolate; dUMP, deoxyuridine 5′-monophosphate; TMP, thiamin monophosphate; Pter., pteroate; Fol., folate; LV, leucovorin; TdR, thymidine; TP, trimethoprim; AP, aminopterin; MT, methotrexate; FdUMP, 5-fluorodeoxyuridine monophosphate.

The screening method is based on the utilization of *Enterococcus faecium* (a species of *Streptococcus* spp.) as the test organism. Fig. 8 summarizes respective folate metabolisms in general microorganisms, *E. faecium* and animal cells, as well as the action sites of such drugs.55) The advantage to use *E. faecium* lied in the fact that this bacillus, different from usual bacteria, lack part of enzyme system for folate biosynthesis, and can incorporate folate-related substances for biosynthesis of purine and pyrimidine bases such as adenine, guanidine and thymine which are the products of C1-transfer by folates, whereas the most general microorganisms having complete enzyme systems cannot incorporate folate-related compounds and stop growing when folate biosynthesis is inhibited. Therefore, in this screening method, it is possible to examine whether a substance is an inhibitor of folate metabolism or not by comparing the difference in antibacterial activities in the presence and absence of a folate-related substance added to the fundamental culture medium. The possible inhibition sites predicted from the inhibition patterns of known compounds are shown in Table V.55),56) For example, in the case of thymidylate synthase inhibitor, the inhibition should be reversed by thymidine, but not by pteroic or folic acid.

**Fig. 9:**
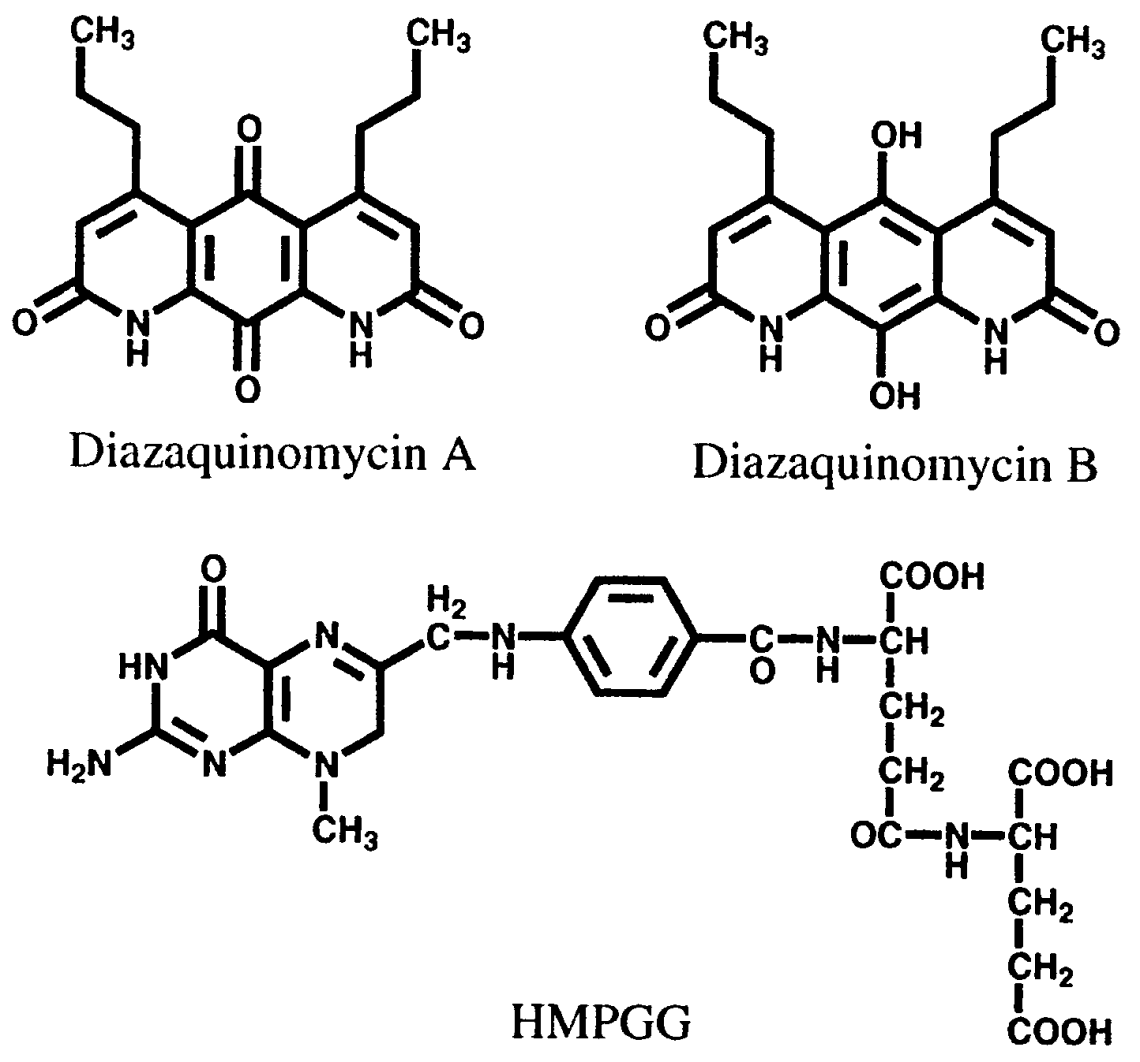
Structures of diazaquinomycins and HMPGG.

Diazaquinomycins A and B,57),58) and 7-hydro-8-methylpteroylglutamylglutamic acid (HMPGG)59) were discovered using the method indicated above (Fig. 9). Diazaquinomycins A and B were isolated from the culture broth of *Streptomyces* sp. Growth inhibition of *E. faecium* by diazaquinimycin A was reversed by the addition of thymidylate, but not by the addition of folate, dihydrofolate or leucovorin to the medium, indicating that the compound is a thymidylate synthase inhibitor.60) This antibiotic exhibited antibacterial activity against Gram-positive bacteria, cytotoxicity against mammalian cell lines, and antitumor activity.57),60),61) HMPGG is structurally closely related to THF and was isolated as an antifolate from a new species of actinomycete. 62) The antibiotic inhibited the growth of *E. faecium*, but was inactive against other bacteria and fungi. The site of inhibition in the folate metabolic pathway was shown to be thymidylate synthase.

### (II) Inhibitors of lipid metabolism

Lipid metabolism is normally maintained in a delicate balance between synthesis and degradation. When that balance is lost, a variety of serious diseases can develop. These include arteriosclerosis (like atherosclerosis), hypertension, obesity, diabetes, functional depression of some organs and so on. Control of lipid metabolism by drugs is expected to be useful against such disorders. Microbial products inhibiting lipid metabolism, particularly those involved in the fatty acid and cholesterol metabolic pathways, were screened using various assay systems by our group, and the methods and results are previously reviewed.63)

#### (a) Acyl-CoA synthetase inhibitors

Long-chain fatty acid-CoA, an activated form of fatty acid, is an important intermediate in lipid biosynthesis, ***β*** -oxidation and in acyl transfer reactions. The conversion of a free long-chain fatty acid to the corresponding acyl-CoA is catalyzed by acyl-CoA synthetase. In animal tissues and cells, this is the main route to supply acyl-CoA, and provides an important target site for drug action to control fatty acid metabolism. On the other hand, fatty acid metabolism in *Candida lipolytica* has been studied extensively. In *C. lipolytica* there are two different acyl-CoA synthetases, distinct in physiological role and localization. Acyl-CoA synthetase I, which is present in microsomes and mitochondria, is solely responsible for cellular lipid biosynthesis, and acyl-CoA synthetase II, which is localized in peroxisomes, provides acyl-CoA that is then degraded via ***β*** -oxidation to yield acetyl-CoA.64)–66)

In our screening program, two mutants of *C. lipolytica* strains, L-7 (defective in acyl-CoA synthase I)64) and A-1 (lacking fatty acid synthase activity),67) were used as test organisms (see Fig 4 in ref. 63)). These mutant strains were grown on two types of media, containing either fatty acid as the sole carbon source or glucose plus a small amount of fatty acid. Possible inhibition sites as predicted from inhibitory patterns are summarized in Table VI.

**Fig. 10:**
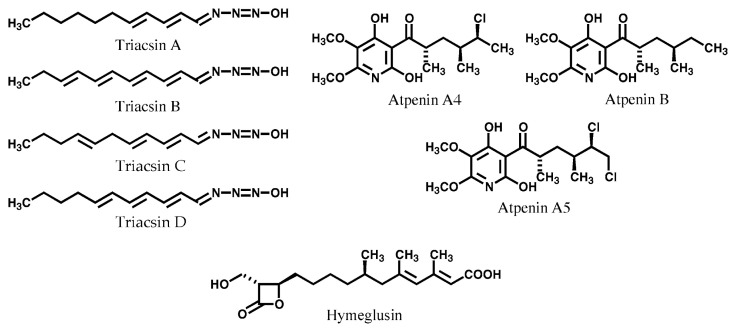
Structures of inhibitors of acyl-CoA synthetase and HMG-CoA synthase.

During the course of this screening program, triacsins A–D were isolated from the culture broth of a *Streptomyces* strain.68),69) They showed inhibitory activity against strain A-1 grown in the two types of media but no effect was seen on strain L-7 in either medium, suggesting that the inhibition site may be acyl-CoA synthetase I. The structures are shown in Fig. 10. The effect of triacsins on bacterial- and animal-derived acyl-CoA synthetases were examined (Table VII).68) Among the triacsins A–D, triacsin C (identical to WS-1228 A70)) was the most potent, and A was the second most potent. Triacsins B and D (identical with WS-1233 B70)) showed much lower activities. The inhibition appeared to be specific to the long-chain fatty acids. Arachidonoyl-CoA formation from mouse fibrosarcoma cells is also sensitive to triacsin action.71) One of the interesting biological characteristics of triacsins is that triacsins show no antimicrobial activity but exhibit potent growth inhibition against animal cells. It has been demonstrated that acyl-CoA synthetase is essential in animal cells for supplying acyl-CoA, but an additional pathway exists in bacteria, yeast and filamentous fungi.72) Triacsins, unique inhibitors of acyl-CoA synthetase, are being applied to the field of lipid research as biochemical tools.72),73)

In the course of this screening program, new antifungal antibiotics atpenins, were isolated from *Penicillium* sp. (Fig. 10).74),75) Atpenins inhibit the growth of both mutant strains A-1 and L-7 of *C. lipolytica*, and also inhibit the incorporation of long-chain fatty acids into the lipid fraction of Raji cells. Studies on the action site of atpenin B revealed that the cellular ATP level in Raji cells was reduced dramatically in the presence of atpenin B, suggesting the inhibition of ATP-generating system by this antibiotic.76) Recently it was found that atpenins potently and specifically inhibit the complex II (succinate-ubiquinone reductase) of mitochondria. 77) Therefore atpenins, specially atpenin A5, may be ideal tools to further study both the role of complex II in various cellular and physiological processes and the mechanisms of electron transfer in mitochondrial complex II.

#### (b) HMG-CoA synthase inhibitor

**Fig. 11:**
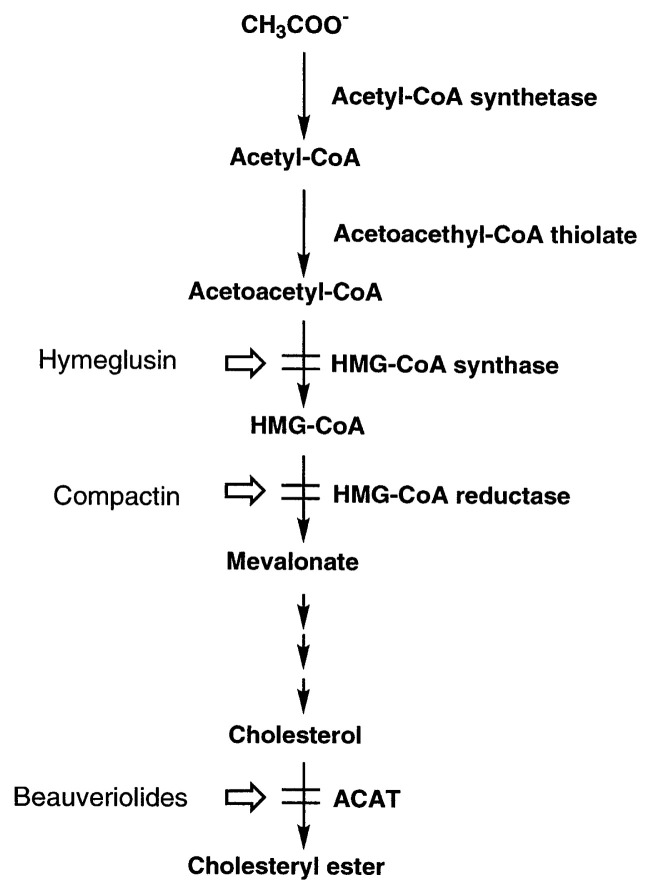
Biosynthetic route leading to cholesterylester via mevalonate pathway and inhibition sites of hymeglusin, compactin and beauveriolides. HMG, 3-hydroxy-3-methylglutaryl; ACAT, acyl-CoA:cholesterol acyltransferase.

Since the discovery of an inhibitor of cholesterogenesis, fungal metabolite ML-236B78) (identical with compactin79)), much attention has been paid to the inhibitors of mevalonate pathway as potential hypocholestrolemic agents. Mevalonate is a key intermediate in cholesterol biosynthesis. It is produced from acetyl-CoA through three enzymatic steps, namely, acetoacetyl-CoA thiolase, 3-hydroxy-3-methylglutaryl-CoA (HMG-CoA) synthase and HMG-CoA reductase (Fig. 11). These enzymes provide promissing targets for hypocholesterolemic agents. Indeed, inhibitors of HMG-CoA reductase, pravastatin (a derivative of ML-236B) and simvastatin (analog of ML236B), have been developed and commercialized as hypocholesterolemic agents.

It was reported that ML-236B inhibits the growth of cultured animal cells and that the inhibition was overcome by the addition of mevalonate to the medium.80) Our screening program utilized Vero cells for primary screening, since the cells are sensitive to ML-236B and both morphological changes and growth inhibition was reversed by addition of mevalonate. The candidate microbial cultures showing growth inhibition of Vero cells and reversion of the inhibition by the addition of mevalonate were selected and active principles were searched.

Using this procedure, a ***β*** -lactone compound designated hymeglusin (1233A) (Fig. 10) was discovered as the product of a *Scopuraliopsis* strain.81)–83) This compound was originally isolated as an antifungal antibiotic from *Cephalosporium* sp. by Aldridge *et al.*84) Hymeglusin was tested on three enzymes, and it was found to specifically and potently inhibit HMG-CoA synthase.85) Thus, the compound became the first HMG-CoA synthase inhibitor of microbial origin (Fig. 11).

#### (c) Macrophage foam cell formation inhibitors

In the early stage of atherosclerosis, macrophages penetrate into the itima and efficiently take up modified low-density proteins (LDL) to store cholesterol as cholesteryl ester (CE) and fatty acids as triglycerides (TG) in the cytosolic lipid droplets. Such macrophages are then converted into foam cells (cells full of lipid droplets), leading to the development of atherosclerosis in the arterial wall. Therefore, inhibitors of macrophage-derived foam cell formation were expected to retard the progression of atherosclerosis.86)–89) Nishikawa *et al.* developed a foam cell formation model using primary mouse peritoneal macrophages cultured in the presence of lipid liposomes containing phospholipids.90) In this cell assay, it is possible to observe the macrophage-derived foam cell formation.

Based on the above observation, we developed a cell-based assay system consisting of microscopic observation of oil red O-stained lipid droplets formation (morphological assay: see Fig 17 in ref. 63)) and measurement of [^14^C]neutral lipids (CE and TG) syntheses from [^14^C]oleic acid (biochemical assay).91) For the primary screen, culture broths that caused reduction of the sizes and/or the numbers of lipid droplets without cytotoxic effect on macrophages were selected. Then, the inhibition was confirmed by biochemical assay as the secondary step.

**Fig. 12:**
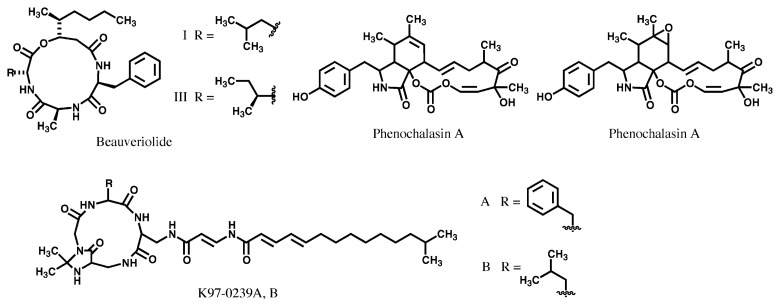
Structures of inhibitors of lipid droplet formation in macrophages.

With this screening system, beauveriolides I and III from a *Beauveria* strain,92)–94) phenochalasins A and B from a *Phomopsis* strain,95),96) and K97-0239s A and B from a *Streptomyces* strain97) were isolated (structures are shown in Fig. 12). Beauveriolides I and III caused reductions in the numbers and sizes of cytosolic lipid droplets in macrophages at 10 μM without any cytotoxic effect on macrophages. They inhibited [^14^C]CE synthesis specifically with IC_50_ values of 0.8 and 0.4 μM, respectively. Studies on the mode of action revealed that they inhibited acyl-CoA: cholesterol acyltransferase (ACAT) activity in microsomes prepared from both mouse macrophages and mouse liver (see Fig 10 in ref. 63)), suggesting that beauveriolides inhibit both ACAT-1 and -2 isozymes to similar extents. Recent research on mammalian ACAT has shown that the two isozymes play different roles in the body.98) ACAT-1 contributes greatly to macrophage-derived foam cell formation in atherosclerosis and ACAT-2 is responsible for absorption of dietary cholesterol from the intestine. Moreover beauveriolides I and III exert antiatherogenic activity in both low-density lipoprotein receptor- and apolipoprotein E-knockout mice without any side effect such as diarrhea or cytotoxicity to adrenal tissues as observed for many synthetic ACAT inhibitors.99)

Both phenochalasins and K97-0239s inhibited lipid droplet formation in macrophages and CE synthesis.

We have tested the effects of several known inhibitors of lipid metabolism using this screening system. Among them, triacsin C (an inhibitor of long chain acyl-CoA synthetase shown above) was found to inhibit both [^14^C]CE and [^14^C]TG syntheses with similar IC_50_ values of 0.19 and 0.10 μM, respectively, and to inhibit the mouse macrophage-derived foam cell formation almost completely at a concentration of 0.59 μM.92) The process of cytosolic lipid droplet formation in macrophages and potential targets of microbial inhibitors are schematically summarized as shown in Fig. 16 of ref. 63). We are planning to test beauveriolides and triacsin C in LDL receptor-deficient mice, an *in vivo* model of atherosclerosis.

## Screening for inhibitors of other functional proteins

We applied cell-based screening systems to the detection of inhibitors of functional proteins other than enzyme systems, and also discovered several novel microbial metabolites. Among these, two kinds of compounds with unique structures and activities are introduced in this review; madindolins to inhibit the functions of interleukins 6 and 11, and lactacystin as an inducer of neurite outgrowth of Neuro 2a cells (a mouse neuroblastoma cell lines).

### (I) Interleukin 6 inhibitors

It is well-known that cytokines not only contribute to homeostasis via immune responses and biological defense, but that they are also involved in cancer, inflamation, allergies, and autoimmune diseases.100) Interleukin 6 (IL-6) is a multi-functional cytokine involved in the control of antibody production, T cell activation, hematopoiesis, acute responses and the growth of certain types of tumor cells.101),102) However, overproduction of IL-6 is closely associated with cancer cachexia, Castleman’s disease, rheumatoid arthritis, hypercalcemia and multiple myeloma.103),104) Since no effective therapeutic drug is currently available for these diseases, inhibitors or modulators of this cytokine functions by new action mechanisms should be developed for therapeutic use for these diseases.

**Fig. 13:**
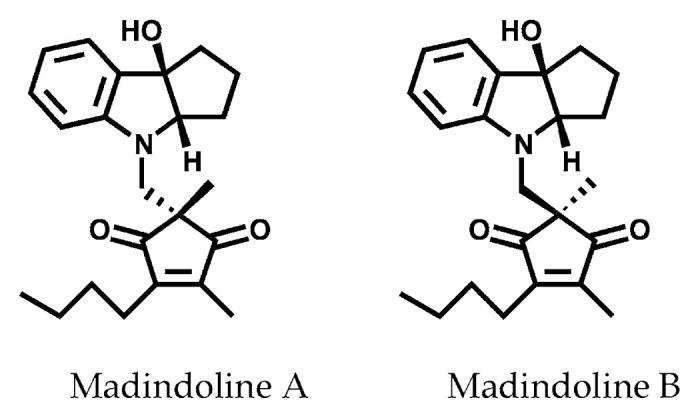
Structures of madindolines A and B.

We screened inhibitors of IL-6 functions using IL-6 dependent MH60 cells, murine hybridomas established by fusing mouse B cells with mouse P3U1myeloma cells.105) Candidate culture broths showing growth inhibition against IL-6 dependent MH60 cells but no activity against IL-6 independent cells were selected and active compounds were searched. A *Streptomyces* strain was found to produce active metabolites designated madindolines A (MDL-A) and B (MDL-B),106) which are stereoisomers consisting of a hydroxyindoline and a diketocyclopentene moieties.107) The absolute stereochemistries of these compounds were established by a total synthesis (Fig. 13).108)

**Fig. 14:**
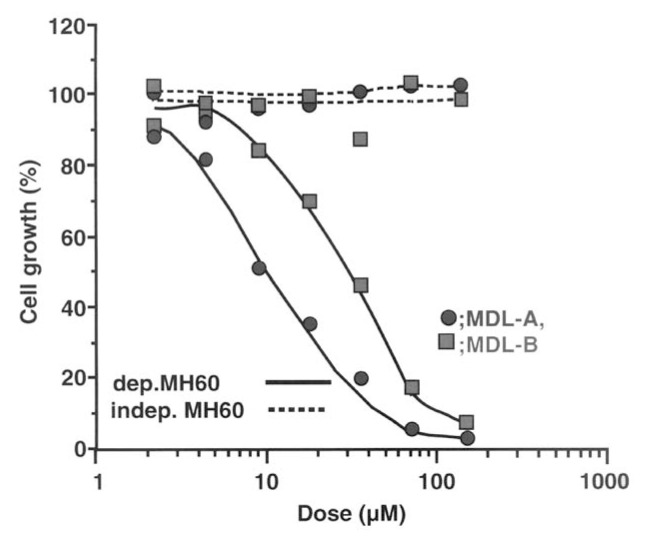
Effect of madindoline A (MDL-A) and B (MDL-B) on the growth of IL-6 dependent and independent MH60 cells. ● MDL-A, ■MDL-B, —MDL-A and B treated IL-6 dependent MH60 cells, ---- MDL-A and B treated IL-6 independent MH60 cells.

**Fig. 15:**
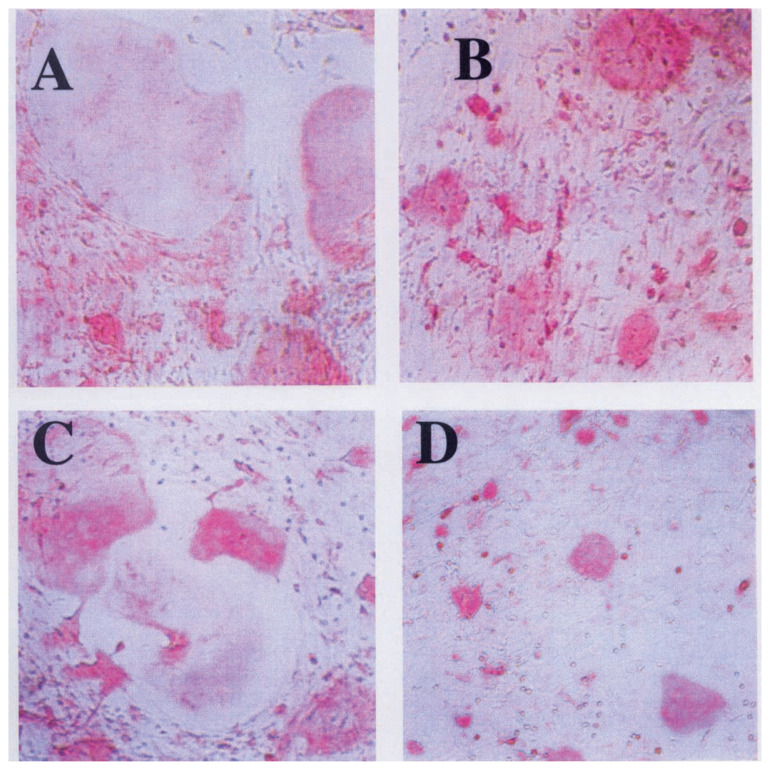
Inhibitory effect of madindoline A on IL-6 and IL-11 induced differentiation of osteoblast to osteoclast. A) IL-6 50 U/ml + sIL-6R 50 ng/ml; B) IL-6 50 U/ml + sIL-6R 50 ng/ml + MDL-A 50 ng/ml; C) IL-11 40 ng/ml; D) IL-11 40 ng/ml + MDL-A 50 μg/ml.

MDL-A and -B specifically inhibited growth of IL-6 dependent MH60 cells in a dose-dependent manner with IC_50_ values of 8.0 and 30 μM, respectively, but they did not affect IL-6 independent MH60 cells (Fig. 14).106) To examine the effect of MDL-A on cytokine activities, the effect on other cytokines were also tested (Table VIII). MDL-A inhibited IL-6 dependent cell growth, and IL-6 and IL-11 mediated activities, but showed no effects on cell growth and cellular functions caused by other cytokines tested. The result indicated that madindolines are specific inhibitors of IL-6 and IL-11 functions.109) Figure 15 shows inhibitory effect of madindolin A on the cytokine-induced differentiation of osteoblast to osteoclast.

**Fig. 16:**
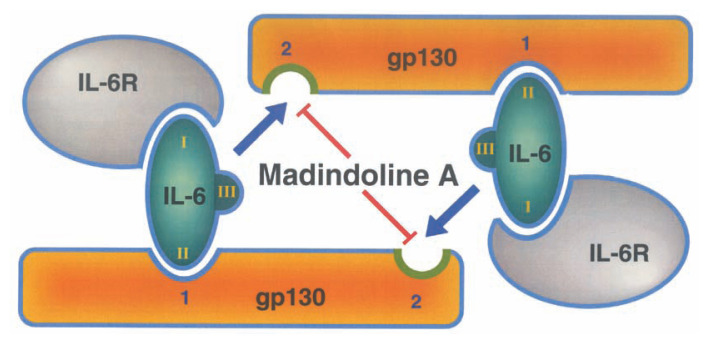
Inhibition mode of madindoline A by binding to gp130.

Subsequently, potential target of madindolines in IL-6 functions was studied.110) The IL-6 receptor system consists of two components, ligand binding gp80 (***α*** chain, an IL-6 specific subunit) and signal transducing gp130 (***β*** chain, common to IL-6 receptor family). As shown above, MDL-A inhibited the functions of both IL-6 and IL-11 without affecting the IL-6-specific signal transduction cascade, JAK2/STAT3, and this suggests that the inhibition site of madindolines might be gp130. In fact, [^3^H]MDL-A bound to gp130 in a dose-dependent manner. Other IL-6 type cytokines such as leukemia inhibitory factor (LIF), oncostatin M (OM), chilary neurotrophic factor (CNTF) and cardiotrophin 1 (CT-1) share gp130 as a common subunit. However, LIF-induced macrophage differentiation was not inhibited by MDL-A. It is noted that IL-6 and IL-11 receptors act through the homodimerization of gp130, whereas the others require heterodimerization of a specific receptor and gp130. Based on these findings, we concluded that the target molecule of MDL-A is gp130, and the compound inhibits the homodimerization of the activated gp130, conceivably via covalent modification of one (or both) of the free cysteine residues (Cys279 and Cys469) that are hypothesized to form a disulfide during cytokine activation (Fig. 16).111)

MDL-A markedly inhibited not only IL-6- and IL-11- stimulated osteoclastogenesis *in vitro*, but IL-6-stimulated serum amyloyd A production and bone resorption in an experimental model of postmenopausal osteoporosis *in vivo* by a mechanism different from that of 17***β*** -estradiol. Since MDL-A has no cytotoxic activity, this is expected to serve as a lead compound for the development of new drugs for treating and preventing hormone-dependent post menopausal osteoporosis, as well as other refractory diseases known to involve IL-6.

### (II) Proteasome inhibitor

Peptide neurotrophic factors (NTF), including nerve growth factor (NGF), are known to be essential for the survival and functional maintenance of nerve cells, and have attracted attention due to the increase in the number of patients with diseases of the nervous system, including senile dementia such as Alzheimer’s disease. Through our study of “motilides”, erythromycin derivatives that show gastrointestinal motilin-like activity without antimicrobial activity,112) the idea arose that microorganisms may produce small molecules with peptide hormone-like activities. Furthermore, nonpeptide small molecules including gangliosides were reported to induce differentiation of neuroblastoma cells, and resulted morphological and biochemical alterations that are very similar to those of NTF-treated nerve cells.113) These facts led us to speculate that small molecule having NTF-like activities are expected to be useful for the medical treatment of nervous diseases.

**Fig. 17:**
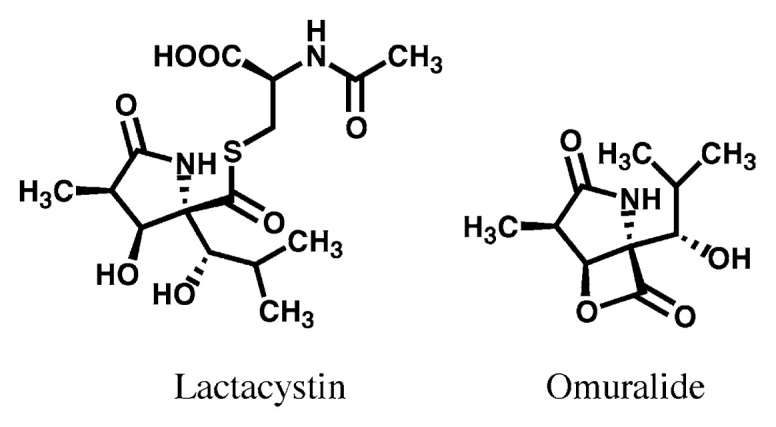
Structures of lactacystin and omuralide (active form of lactacystin).

Microbial culture broths were assayed to screen for compounds that induce differentiation of the mouse neuroblastoma cell line Neuro 2a. According to this screening procedure, a novel *γ* -lactam compound designated lactacystin was isolated from a *Streptomyces* strain.114),115) The structure consists of two amino acids, (*R*)-N-acetylcysteine and a novel ***α***-substituted pyroglutamic acid, which are connected via a thioester linkage (Fig. 17).

**Fig. 18:**
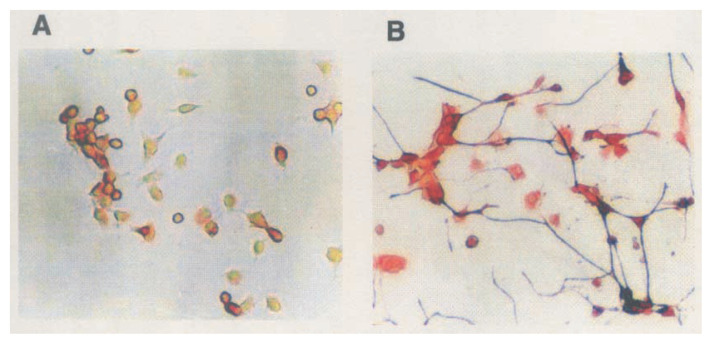
Immunofluorescence staining of 200 kDa neurofilament in Neuro 2A cells. The cells cultured were plated at a density of 1.5 × 10^5^ cells/60 mm i.d. dish and incubated at 37 °C. Following photomicrographs are: (A) Control (after 4 days); (B) 4 days after lactacystin (1,3 μM) treatment. The control cells remained in compact form and exhibited minimal neurite extention. Most of cells responded to 4-day treatment of lactacystin by elaborating long neurites.

Lactacystin treatment caused characteristic changes in the morphology of Neuro 2a cells. It elicited the formation of short solid bipolar projections from both sides of the cell body after one day, and the cells became spherical like control cells, disappearing neurites on day 2. Prolonged treatment (on day 4) led to hetrogeneous morphology, and neurite networks were formed (Fig. 18).114) Lactacystin induced morphological changes of the Neuro 2a cells in dose-dependent manner at concentrations higher than 0.22 μM, but it showed cytotoxicity was observed at above 5.2 μM. The morphological changes induced by lactacystin differ from those induced by other neurite-inducing compounds. 116) These findings as well as other biochemical features indicate that the mechanism by which lactacystin induces neuroblast cell differentiation differs from those of other differentiation inducing factors. This is the first microbial metabolite to exhibit neurotrophic activity, and was expected to be used as a useful tool for investigations of differentiation mechanism of the nervous system. A study on the mode of action of lactacystin in neuritogenesis revealed that it arrests the cell cycle in Neuro 2a cells at both G0/G1 and G2 phases. However, it showed no effect on the enzymes responsible for cell cycle such as protein kinases and histone deacetylase. These data suggest that lactacystin has target molecule(s) other than enzymes shown above.117)

**Fig. 19:**
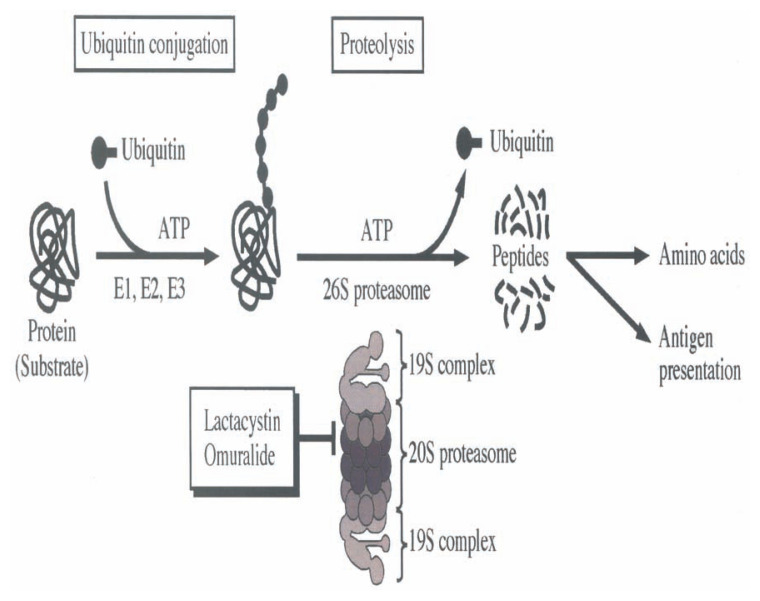
The ubiquitin-proteasome pathway for protein degradation. The protein substrate is first polyubiquitinated in a reaction involving ubiquitin-activating enzyme (E1), ubiquitin-conjugating enzyme (E2) and ubiquitin-protein ligase (E3). The polyubiquitinated substrate is rapidly hydrolyzed by 26S proteasome containing the core 20S proteasome enclosed by two 19S reguratory complexes. Deubiquitinating enzyme allows the recycling of ubiquitin molecules. Most of the peptide products are hydrolyzed into amino acids by exopeptidases.

The groups of Corey and Schreiber studied on the target molecules of lactacystin using the sample offered by us and synthetic analogs. In 1995, they reported that lactacystin inhibits the function of proteasome which is a large, multicatalytic protease complex responsible for most non-lysosomal intracellular protein degradation. 118) In this proteolytic pathway, most substrates are marked by conjugation of multiple ubiquitin molecules to be degraded rapidly by the 26S proteasome (2000 kDa).119),120) The 26S proteasome consists of a 20S proteasome core (700 kDa) which carry the catalytic center, and two 19S complexes (Fig. 19).

**Fig. 20:**
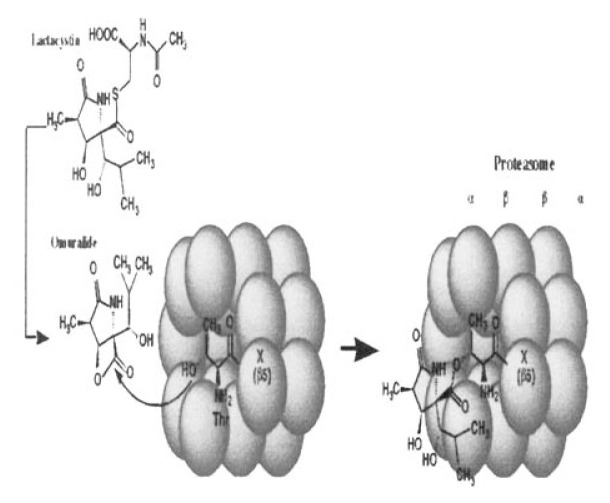
Mechanism of lactacystin binding to NH_2_ terminal threonine, an active center of X(***β*** 5) subunit in proteasome. Lactacystin is converted to the active and cell-permiable form omuralide in aqueous solution. The ***β*** -lactone ring of omuralide takes nucleophilic attack by the hydroxy group of the NH_2_ terminal threonine, resulting covalent modification of an active site in proteasome.

Synthetic studies to clarify structure-activity relationships of lactacystin analogs have been performed by Corey-Schreiber group121) and our group.122) Based on the evidence that lactacystin ***β*** -lactone (shown in Fig. 17 and designated as “omuralide” by Corey123)) shows activity similar to lactacystin together with kinetic analysis of its inhibition, lactacystin is recognized to serve as a prodrug for proteasome inhibition. Namely, lactacystin is transformed to the reactive ***β*** -lactone, and this cell-permeable transformant rapidly enters into the cells and reacts with the target residue. Groll *et al.* reported the X-ray crystallographic analyses of the yeast 20S proteasome-lactacystin complex, confirming that lactacystin is covalently bound to the N-terminal threonine residue of the ***β*** -type subunit PRE-2 in 20S core corresponding to the human subunit X (Fig. 20).124) Lactacystin and its active transformant (omuralide) are now recognized as specific inhibitors of proteasome. They have been utilized to investigate proteasome functions, and the results obtained from these studies have clarified the importance of cellular proteasome functions and ubiquitin-proteasome pathway.125),126)

On the other hand, it was shown that lactacystin effectively prevented etoposide resistance *in vitro* in both resistant and sensitive tumor cells (colon cancer HT-29 and ovarian cancer A2780 cells, respectively).127) Previous work demonstrated that stress conditions induce decreased expression of DNA topoisomerase II***α*** (topo II***α***),128) rendering cells resistant to topo II-targeted drugs, such as etoposide and doxorubicin. Our present study showed that lactacystin effectively blocked topo II***α*** protein depletion occurred through proteasome-mediated degradation mechanisms. This study indicates that the specific proteasome inhibitors, lactacystin and omuralide, may be useful for improving the efficacy of topo II-targeted chemotherapy against solid tumors.

## Conclusion

More than 20,000 compounds of microbial origin have been reported in the literature.1),2) The fact suggests that microorganism are a superior source of metabolic diversity. The search for bioactive microbial metabolites is one of the central themes in gaining access to the outstanding molecular diversity available in nature for drug development. In order to achieve success in new drug discovery from microbial sources, various screening strategies like target-directed, biological, physicochemical, or chemical methods have been attempted.6)–8),129),130)

In this review, we introduce the results obtained in our group from cell-based screening methods using intact microbial and mammalian cells having various functions. Some of the advantages of cell-based screening strategies have been demonstrated. Phosalacine itself was almost inactive against glutamine synthetase *in vitro*, but the compound was detected by our screening method because of easy conversion to an active form, phosphinothricin, in bacterial cells. Similarly, lactacystin isolated as a NTF-like agent was incidentally found to serve as a prodrug of a specific inhibitor of proteasome. This compound is transformed to the reactive ***β*** -lactone “omuralide” that bind to the target residue in proteasome. Another merit was shown to be the reduction of false positives. Though it was reported that arachidonate and linolate inhibit HMG-CoA synthase in an *in vitro* enzyme assay,131) they were hardly expected to show *in vivo* efficacy. In our screening for mevalonate inhibitors, inhibition of Vero cell growth by arachidonate was not reversed by the addition of mevalonate, and linolate showed no effect on Vero cell growth,83) indicating that the false positive results due to fatty acids could be eliminated.

Cell-based screening methods are not only limited to exploitation of specific functions of special cells. Screening to detect activities that induce morphological abnormalities of microbial and animal cells can provide various types of information on the test samples. Beppu *et al.* attempted to screen for antifungal antibiotics based on the induction of morphological deformation of filamentous fungi and yeast,132) and identified leptomycins, which cause cell elongation of *Schizosaccharomyces* and hyphal swelling or curling of *Mucor* spp. These compounds induce cell cycle arrest. Iwasaki (one of the authors of this review) *et al.* found fusarielins according to characteristic hyphal curling of a *Pyricuralia oryzae* strain.133) This kind of morphological deformation was originally observed by treatment with rhizoxin, a specific microtubule inhibitor, and the phenomenon has been utilized as a detection method for anti-microtubule agents.

Based on the results summarized in this review, it is evident that the cell-based screening methods are useful for finding active principles with selective modes of actions, and the strategies shall be developed further.

## Figures and Tables

**Fig. 4:**
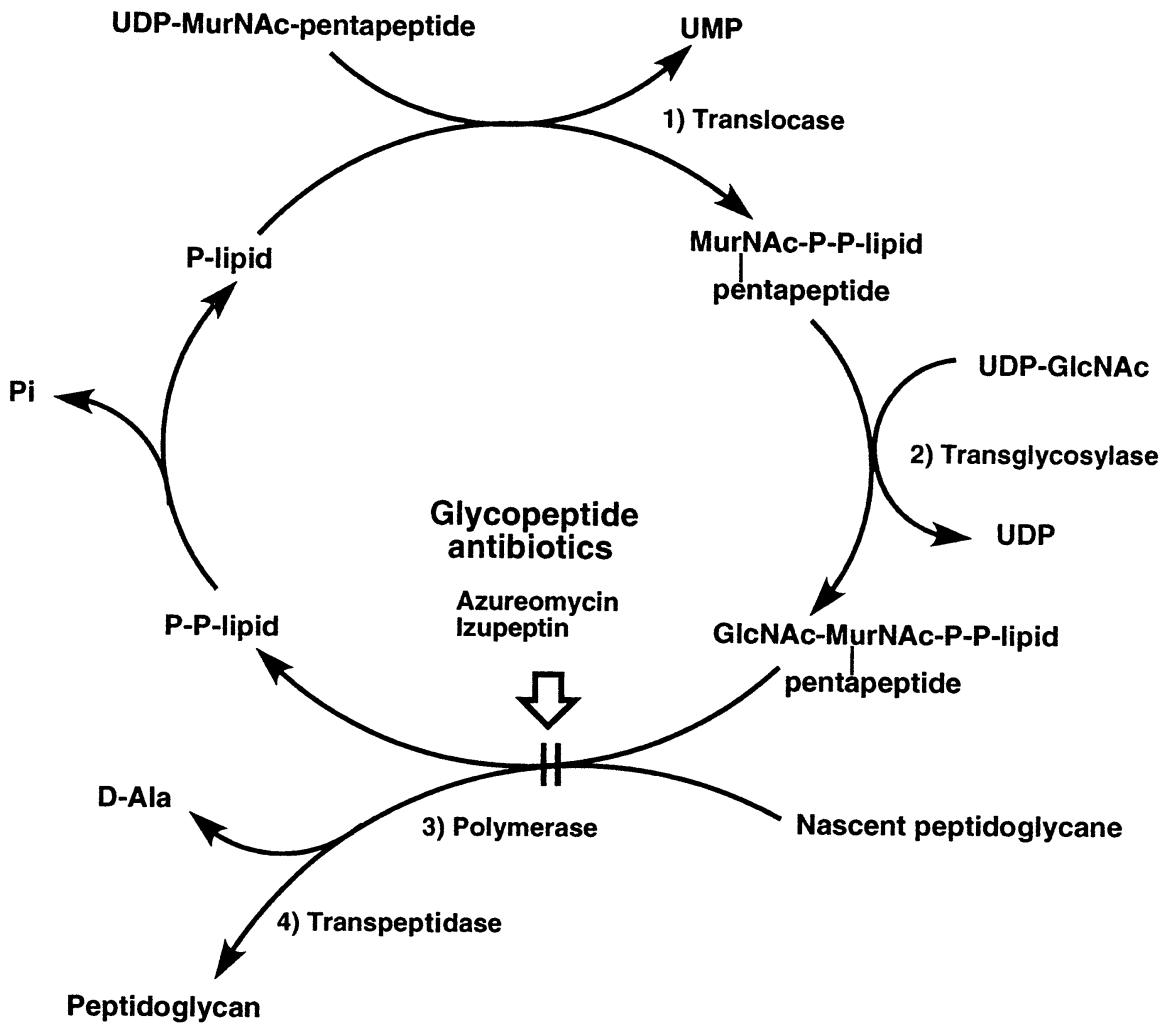
Action site of glycopeptide antibiotics in the peptidoglycan biosynthetic pathway of *B. megaterium* KM. UDP, uridine diphosphate; UMP, uridine monophosphate.

**Fig. 5:**
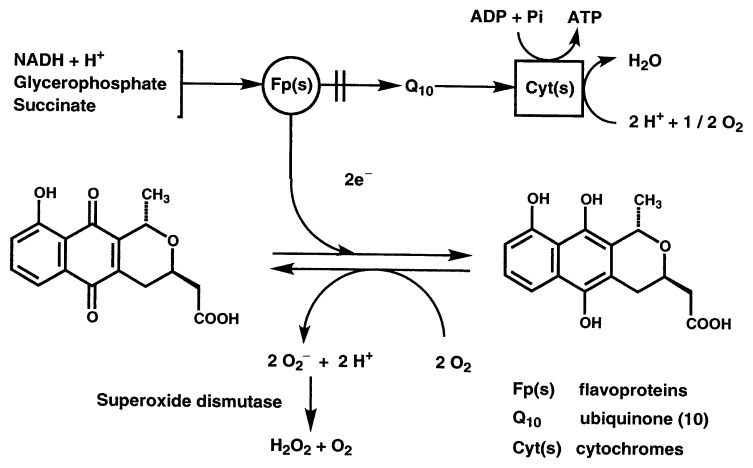
Proposed mechanism of action of nanaomycin A (or D) in *Vibrio alginolyticus*. ADP, adenosine diphosphate; ATP, adenosine triphosphate; NADH, nicotinamide adenine dinucleotide reduced form.

**Table I:** Microbial metabolites used as medicines and/or biological reagents, discovered by S. Ōmura and his co-workers (by 2001)

Used as medicines		Used as biological reagents	
	
Compound	Biological activity	Compound	Biological activity
	
avermectin	anthelmintic (for animal and human)	cerulenin	fatty acid and polyketide synthase inhibitor
frenolicin B	antimycoplasma (for chickens)	herbimycin A	tyrosine kinase inhibitor inhibitor of HSP90 function
leucomycin A_3_	antimicrobial (for human)	lactacystin setamycin (bafilomycin B_1_)	proteasome inhibitor vacuolar ATPase inhibitor
nanaomycin A	antimicrobial (for animal)	staurosporine triacsin	protein kinase inhibitor acyl-CoA synthetase inhibitor
rokitamycin	antimicrobial (for human)	vineomycin B_2_	collagen prolyl hydroxylase inhibitor
tilmicosin	antimicrobial (for animal)	atpenin	complex II inhibitor (in succinate-quinone reductase)

**Table II:** Anitimicrobial activities of nanaomycin A and B

Test organism	Medium^a^	Minimum inhibitory concn (−g/ml)	

		A	B
*Candida albicans* KF-1	P	31.2	31.2
*Saccharomyces sake* KF-26	P	31.2	62.5
*Aspergillus niger* ATCC 6275	P	62.5	62.5
*Pyricularia oryzae* KF-180	P	7.8	15.6
*Trichophyton asteroides* KF-50	P	1.6	12.5
*T. mentagrophytes* KF-213	P	0.8	25
*T. purpureum* KF-61	P	3.1	25
*T. rubrum* KF-53	P	0.1	3.1
*Mycoplasma gallisepticum* KP-13	H	<0.013	<0.013
*M. gallisepticum* 333P	H	<0.013	<0.013
*M. gallinarum*	H	1.56	3.12
*M. pneumoniae* KB-173	E	0.013	3.05
*Acholeplasma laidlawii* (B) Bml	H	25	25

a P, Potato agar (pH 6.4, 4 days, 27 °C); H, Hokken PPLO agar (pH 7.8, 8 days, 37 °C); E, Eiken PPLO agar (pH 7.8, 8 days, 37 °C).

**Table III:** Inhibitory pattern and screening system for glutamine synthetase inhibitors

Culture medium	Pattern			

	A	B	C	D
Davis’ minimal medium	+	−	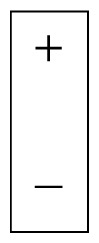	−
Davis’ minimal medium + glutamine	+	−		+

+: Inhibition; −: No inhibition. Pattern C was selected in this screening.

**Table IV:** Tetrahydrofolic acid (THF) derivatives and related metabolic system

THF derivative	C_1_-unit	Related metabolic system
10-Formyl-THF	10-CHO	Purine nucleotides N-Formylmethionyl-*t*-RNA
5,10-Methenyl-THF	5,10-CH=	Purine nucleotides
5,10-Methylene-THF	5,10-CH_2_-	Glycine, serine Pyrimidine nucleotides t-RNA nucleotides
5-Formimino-THF	5-CH=NH	Histidine Purine bases
5-Methyl-THF	5-CH_3_	Methionine
5-Formyl-THF	5-CHO	Histidine

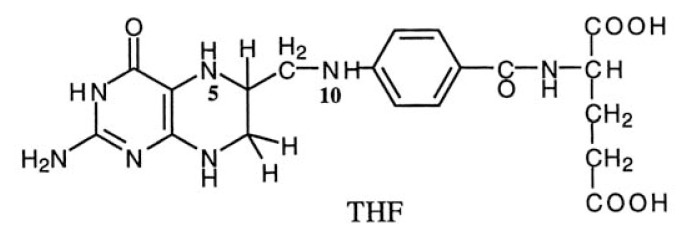		

**Table V:** Antibacterial activities of antifolates against *E. faecium* in media supplemented with folate-related compounds as growth factor*

Site of inhibition	Drugs	Growth inhibition against *E. faecium*				

		Pteroate (1.0 ng/ml)	Folate (1.0 ng/ml)	DHF (1.0 ng/ml)	Luecovorin (1.0 ng/ml)	TdR (1.0 μg/ml)
DHP synthase	Sulfa drug	−	−	−	−	−
DHP reductase	TP, AP, MT	+	+	+	−	−
TMP synthase	5-FU (FdUMP)	+	+	+	+	−

* For abbreviations, see legend to Fig. 8.

−, Inactive; +, active.

**Table VI:** Inhibition site in fatty acid metabolism predicted from growth inhibitory patterns against *Candida lipolytica* mutant strains A-1 and L-7 in a medium containing fatty acid or glucose as a sole carbon source

Possible inhibition site	Inhibitory pattern			

	Mutant A-1		Mutant L-7	
	
	Fatty acid	Glucose*	Fatty acid	Glucose*
Acyl-CoA synthetase I	+	+	−	−
Acyl-CoA synthetase II or ***β*** -oxidation	+	−	+	−
Fatty acid synthase	−	−	+	+

* A small amount of fatty acid (0.01%) was added.

+, Growth inhibition; −, no inhibition.

**Table VII:** Effect of triacsins on acyl-CoA synthetases and acetyl-CoA synthetase

Enzyme source	IC_50_ (μM)			

	Triacsin A	Triacsin B	Triacsin C	Triacsin D
Acyl-CoA synthetase				
*Pseudomonas fraji*	26	>150	17	>150
*Pseudomonas aeruginosa*	17	>200	3.6	>200
Rat liver	18	>200	8.7	>200
Raji cells	12	>200	6.3	>200
*Candida lipolytica*				
ACS-I	5.5	>200	4.0	>200
ACS-II	>>200	>>200	>>200	>>200
HSDMICI cells*				
Long chain ACS	ND	ND	0.48	ND
Arachidonoyl-CoA synthetase	ND	ND	8.5	ND
Acetyl-CoA synthetase				
*Saccharomyces cerevisiae*	–	ND	–	ND

* Mouse fibrosarcoma cells [10].

>, 40–50% inhibition at the indicated concentration; >>, 20–40% inhibition at the indicated concentration; –, no inhibition at 200μM; ND, not determined.

**Table VIII:** The effect of madindoline A on cytokine activities

Cytokine	Cell line	Activity	Inhibition
IL-2	CTLL-2	Growth	−
IL-3	Baf/G-CSFR-gp130	Growth	−
IL-4	U937	Expression (Fc***ɛ***RII)	−
IL-6	MH60	Growth	+
	M1	Differentiation (M***φ***)	+
	PC12	Neuronal differentiation	+
	3T3L1	Differentiation (adipocite)	+
	Osteoblast	Differentiation (osteoclast)	+
IL-8	PMNLs	Chemotaxis	−
IL-11	Osteoblast	Differentiation (osteoclast)	+
	3T3L1	Differentiation (adipocite)	+
TNF	L929	Growth inhibition	−
	U937	Growth inhibition	−
NGF	PC12	Neuronal differentiation	−
G-CSF	Baf/G-CSFR-gp130	Growth	−

−, No inhibition; +, inhibition.
